# Usability and Acceptability of a Mobile Food Record in Free-Living Athletes: A Single-Group Observational Study

**DOI:** 10.3390/nu18193208

**Published:** 2026-09-28

**Authors:** Fabricio Forchino, Dale W. Chapman, Carly J. Brade, Kagan J. Ducker, Fengqing Zhu, Deborah A. Kerr

**Affiliations:** 1 Curtin School of Allied Health, Curtin University, Bentley, WA 6102, Australia; dale.chapman@curtin.edu.au (D.W.C.); carly.brade@curtin.edu.au (C.J.B.); kagan.ducker@curtin.edu.au (K.J.D.); 2 School of Electrical and Computer Engineering, Purdue University, West Lafayette, IN 47907, USA; zhu0@purdue.edu; 3 Curtin School of Population Health, Curtin University, Bentley, WA 6102, Australia; d.kerr@curtin.edu.au; 4 Curtin Medical Research Institute, Curtin University, Bentley, WA 6102, Australia

**Keywords:** dietary assessment, image-based dietary assessment, food photography, dietary monitoring, sports nutrition, energy intake

## Abstract

**Background/Objectives**: Accurate assessment of energy intake in free-living athletes is constrained by reporting burden, reactivity, and variable adherence. Image-based dietary assessment methods, such as the mobile food record application (mFR™), may partially address these limitations, although their usability and acceptability in athletes have not been determined. This study evaluated the usability and acceptability of the mFR™ app in free-living athletes and described adherence to a four-day recording protocol. **Methods**: Twenty athletes (13 males, 7 females; 25.2 ± 7.6 years; 13 classified as Tier 1, recreationally active, and seven as Tier 2 to 3) were asked to record all food and drink over four consecutive days using the mFR™ app installed on their mobile phone device, capturing before- and after-eating images and including a fiducial marker. Acceptability and usability were assessed via an online questionnaire with Likert-scale items and open-ended questions. Adherence was described as recording at least one eating occasion on each of the four target days and as completion of the questionnaire; no adherence criterion was prespecified. Likert responses were summarised using frequencies and percentages, and free-text responses were analysed descriptively at a semantic level. **Results**: Thirteen participants (65%) recorded at least one eating occasion on all four days and all completed the questionnaire (*n* = 20). Most participants (85%) agreed they ‘found it easy to take photos’ of their food and beverage intake. Remembering to capture images before main meals was perceived as manageable (55% agreement), whereas capturing images before snacks was considered challenging (15% agreement). No problems with the app were reported by 90% of participants, and 80% expressed willingness to use the mFR application again. Half of the participants (50%) reported that recording influenced their dietary behaviour, with qualitative responses describing increased awareness of food choices. **Conclusions**: Usability and acceptability of the mFR™ app were generally rated favourably by participants in this study, whereas adherence declined across the four-day recording period and the completeness of recording within each day could not be established. These findings provide preliminary evidence of acceptability, alongside incomplete adherence and unverified within-day completeness, which require attention before the mFR™ app is used to estimate habitual intake in athletes.

## 1. Introduction

Accurate assessment of dietary intake is fundamental to sports nutrition research and practice. Estimates of energy intake (EI) underpin the evaluation of energy availability (EA), a construct closely associated with athlete health, training adaptation, and performance outcomes [1]. Despite its importance, measuring EI in free-living athletes remains methodologically challenging because accuracy and participant burden are difficult to reconcile in a single method. Dietary assessment methods, including food records and 24 h recalls, are prone to reporting bias, participant burden, and behavioural reactivity, whereby individuals may alter their eating behaviour when aware that their intake is being recorded [2,3,4]. These limitations can be amplified in athletic populations, with athletes exhibiting high energy flux, irregular training schedules, frequent snacking, and regular consumption of sports-specific foods and beverages, all of which complicate accurate dietary reporting [5,6]. Systematic reviews have consistently reported substantial measurement error across dietary assessment methods used with athletes, with under-reporting of EI particularly prevalent [6,7]. Furthermore, EI patterns in athletes often display pronounced day-to-day variability that may be inadequately captured by short-duration dietary assessment [8].

Technology-assisted dietary assessment tools have emerged as promising alternatives to address the challenges of participant burden and reporting error associated with conventional dietary assessment methods. Image-based methods rely on image capture to provide the main source of assessment, thereby reducing the burden on the participant to record and describe their intake as occurs with written dietary records. Image-assisted methods, by contrast, use images as a reference to adjust a separate self-report record such as a 24 h recall, whereas image-based methods use the captured images as the primary record of intake [9]. The mFR™ app is an image-based method, and this distinction is applied consistently throughout. These image-based methods enable real-time capture of eating occasions, which minimises dependence on memory and removes the need for the participant to estimate portion sizes, although under-reporting through forgetfulness or social desirability remains possible [4,10]. Among these, the mobile Food Record (mFR™) app integrates before- and after-meal image capture with a structured user confirmation process and has demonstrated adequate validity and usability in community-dwelling adults [9], and adolescents have expressed willingness to record their intake using this method [11] and in a controlled feeding study [12]. Several studies have assessed the usability of the mFR™ app and the willingness of participants to record with it in various populations [11,13]. However, evidence regarding the usability and acceptability of image-based dietary assessment tools in athlete populations remains limited [2,6,14].

The Technology Assisted Dietary Assessment (TADA™) system consists of the mFR application along with an online platform that extends the capabilities of image-based dietary assessment by integrating computer vision and machine learning techniques to support food identification and portion-size estimation. Although the system is designed for automated processing, the current implementation relies on trained analysts to review participant-captured images until the pipeline is fully automated [15]. Within this system, the mFR™ app serves as the participant-facing interface, enabling real-time image capture of foods and beverages before and after consumption. Recent work has demonstrated adequate group-level validity of technology-assisted dietary assessment methods in controlled feeding conditions in adult populations [12]. However, the practical value of such systems is also shaped by usability and perceived burden, which have been shown to influence user acceptability and method preference [16].

Usability and acceptability of the mFR™ app under free-living conditions in athletes, however, remain unclear. This distinction is particularly important because real-world adherence, usability, and perceived burden may substantially influence data completeness and quality. In athlete populations, characterised by high training demands and variable daily routines, these factors may in turn compromise the accuracy of EI estimates. Participant adherence, protocol implementation and tool usability are the factors that ultimately determine the scalability of dietary assessment tools [2]. Usability and acceptability evaluations of the mFR™ app have been undertaken in non-athlete populations [9,11,17], and the one study that applied an image-based method in athletic individuals was designed to test validity against a concurrent weighed food diary, so that adherence was reported only incidentally and perceived burden was not assessed [14]. Therefore, the aim of this study was to evaluate the usability and acceptability of the mFR™ app in free-living athletes and to describe adherence to a four-day recording protocol.

## 2. Materials and Methods

### 2.1. Study Design

**Figure 1:**
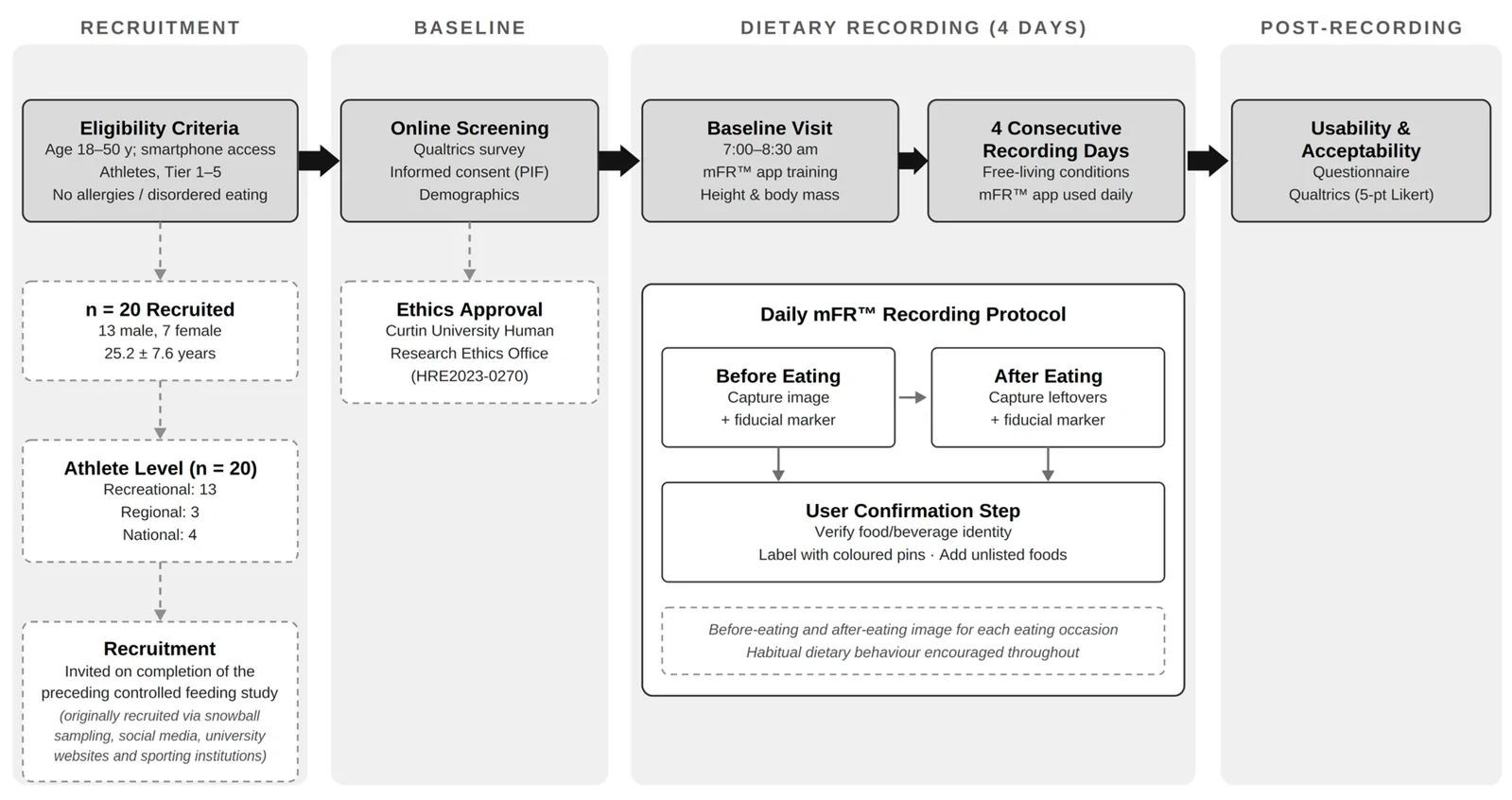
Study design and participant flow. mFR™ = mobile Food Record; PIF = Participant Information Form; TADA™ = Technology-Assisted Dietary Assessment.

This study employed a single-group, prospective observational design to evaluate the usability and acceptability of the mFR™ app in free-living athletes (Figure 1). The study focused on participant adherence, usability, and perceived burden associated with the dietary recording protocol rather than validity or accuracy outcomes. Research was conducted in accordance with the Declaration of Helsinki, and ethical approval was obtained from the Curtin University Human Research Ethics Office (HRE2023-0270).

### 2.2. Participants and Recruitment

Eligible participants were athletes aged 18 to 50 years who were training to compete at a local level or higher (Tier 2–5: Trained/Developmental to World Class) or were actively engaged in training for a minimum of six hours per week, comprising both sport-specific and non-sport-specific training (Tier 1: Recreationally Active) [18]. Additional recruitment criteria required each participant to have access to a smartphone (Apple iOS or Android Operating System), have no dietary restrictions (e.g., known food allergies), and have no self-reported disordered eating. Participants were recruited from athletes who participated in a preceding controlled feeding study of the mFR™ conducted by the same research group, for which a convenience sample had been obtained through snowball sampling, social media advertising on university websites and direct contact with various sporting institutions and groups. On completion of that study, participants were invited to take part in the present study. Potential participants were screened using an online questionnaire (Qualtrics, Provo, UT, USA), which included a brief project introduction and a participant information form. Informed consent was provided by each participant electronically by selecting “I consent to participate in this study” after reading the participant information and informed consent forms. After providing informed consent, each participant answered questions regarding their contact details, demographics, smartphone type, sports characteristics, and dietary restrictions. The 20 athletes who accepted the invitation and provided consent constituted the sample; no formal sample size calculation was undertaken.

### 2.3. Baseline Assessment

Participants attended the Curtin University School of Population Health Laboratory at a pre-scheduled appointment between 7:00 and 8:30 am. During this visit, participants were familiarised with the study procedures, instructed on how to use the mFR™ app, and then proceeded to the clinic room for anthropometric assessment. Body mass and stretch stature were measured in duplicate by a single trained anthropometrist (FF) following the standardised International Society for the Advancement of Kinanthropometry protocol [19], using a column-mounted digital scale (maximum capacity 360 kg; resolution 0.05 kg) and a separate stadiometer (resolution 1 mm), both from the same manufacturer (Seca GmbH & Co. KG, Hamburg, Germany). A third measurement was obtained when the two initial measurements differed by more than 1%, in which case the median of the three values was retained; otherwise, the mean of the two measurements was used. Body mass was recorded with participants in light clothing (t-shirt and shorts) and without shoes. No fasting or activity restrictions were imposed prior to attendance, as anthropometric data were used for sample characterisation rather than as primary outcomes.

### 2.4. Dietary Recording Protocol

**Figure 2:**
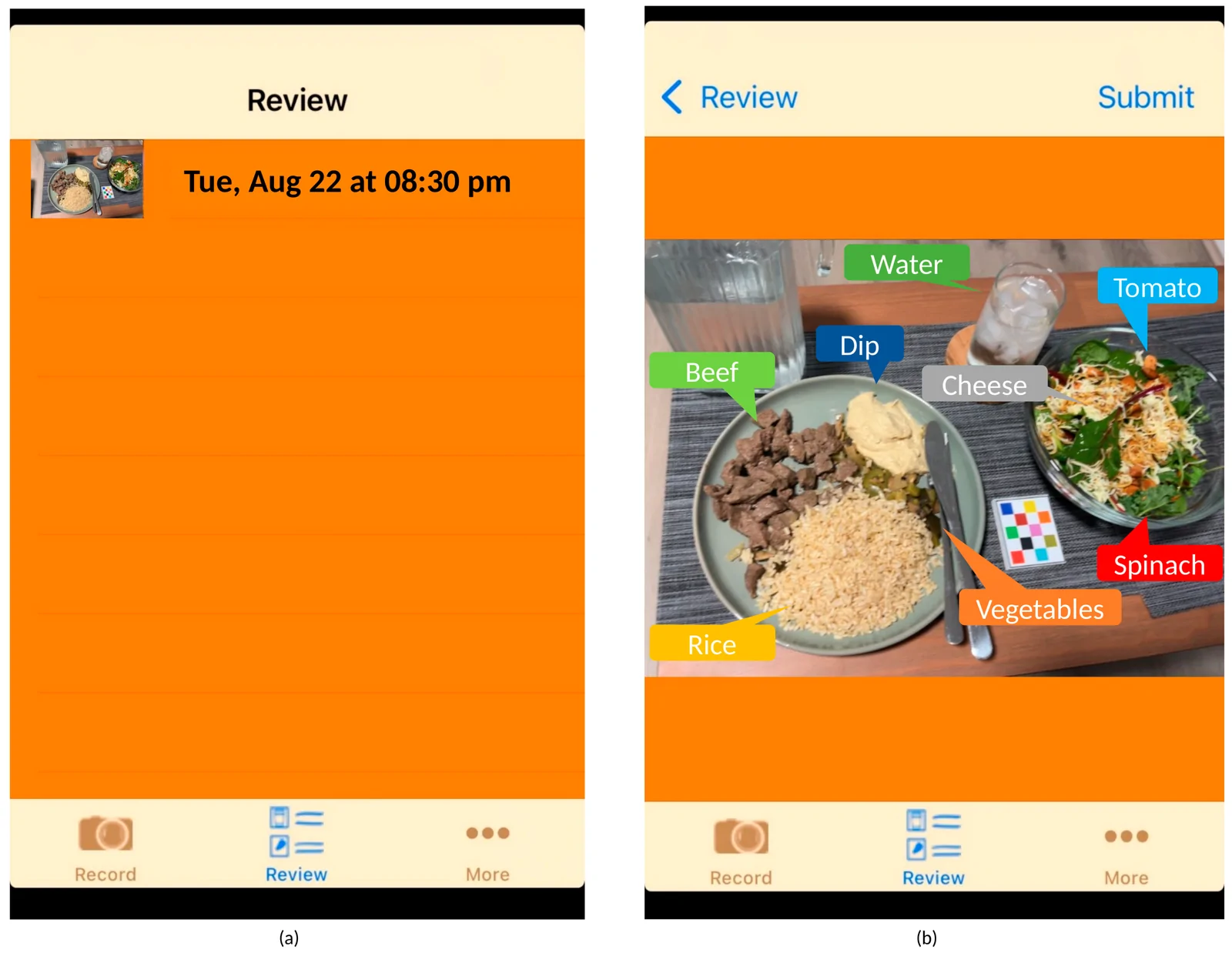
The mFR™ interface during the review process. (**a**) List of captured images, each with its date and time stamp; (**b**) an eating occasion after labelling, in which each food and beverage is identified by a coloured pin and label and the fiducial marker is visible in the image.

Participants were instructed to record all foods and beverages consumed over a target period of four consecutive days using the mFR™ application. The application (mFR™, Purdue University, West Lafayette, IN, USA) was distributed to participants via direct download rather than through an app store and was installed on participants’ own smartphones; version 1.0.0 was used on both operating systems and required iOS 14 or later or Android 5 or later. The recording protocol required participants to capture images of foods and beverages before and after consumption, including any leftovers, where applicable. Participants were instructed to include a fiducial marker, a checkerboard reference object of known dimensions, in each image to facilitate portion-size estimation [15]. Figure 2 illustrates the mFR™ application interface during the image review and labelling process, including the fiducial marker and coloured pin food identification system. Following image capture, participants completed a user confirmation step within the mFR™ app to verify the identity of foods and beverages recorded. Participants were encouraged to maintain habitual dietary behaviours throughout the recording period, which was not standardised with respect to weekdays and weekend days. Within the mFR™ app, an eating occasion was recorded as a pair of images, one captured before eating and one after eating, and only complete pairs were uploaded. The application did not deliver scheduled reminders to record, and no researcher-initiated reminders were used during the recording period. A forgotten before-eating image could be recorded retrospectively through the “forgot image” function, in which the participant entered the food, the time of consumption and an approximate amount, and a forgotten after-eating image could be replaced by an image of the fiducial marker alone.

### 2.5. Acceptability and Usability Assessment

Acceptability and usability were evaluated using an online questionnaire administered via Qualtrics at the end of the four-day dietary recording period (Table 1). The questionnaire was adapted from previously published instruments designed to assess user perceptions, preferences, and behavioural responses to technology-assisted and image-based dietary assessment tools [13,17]. Items addressing the ease of remembering image capture before and after meals and snacks, carriage of the fiducial marker, motivation to use the application and perceived behavioural change were taken from the questionnaire reported by Kerr et al. [13], as were the two open-ended items. The four items related to remembering image capture also corresponded to the prospective form used by Daugherty et al. [17]. Items addressing the device used, image capture, the coloured-pin labelling interface, food-list navigation, the “food not listed” and “forgot image” functions, problems encountered and willingness for future use were adapted from the post-study acceptability questionnaire of the research group’s controlled-feeding evaluation of the current mFR™ implementation [20]. Wording was modified where necessary to refer to the application as presented to participants. The adapted instrument was not pilot tested in athletes, and no formal assessment of content validity or of psychometric properties was undertaken. The full questionnaire is provided as Table S1. Within the questionnaire, the application was referred to as “the TADA app”, which was the name presented to participants at the time of data collection. Item wording is reproduced verbatim in this manuscript and in Table S1, whereas the participant-facing application is referred to as the mFR™ app in all other text, consistent with its designation as the participant-facing interface of the TADA™ system.

Adherence to the recording protocol and completion of the questionnaire were recorded as objective indicators of whether the dietary recording protocol could be implemented in free-living athletes [21]. Acceptability is recognised as a distinct focus of evaluation [21], and it was evaluated here alongside usability as a self-report dimension, the two being distinguished by the object that was evaluated rather than by the perspective from which assessment was undertaken. Usability was operationally defined as the extent to which the athletes could use the mFR™ application to achieve image capture with effectiveness, efficiency and satisfaction in the free-living context of use [22], and was assessed through items addressing image capture, the food-labelling interface, food-list navigation and technical performance. Acceptability was operationally defined as the extent to which the recording procedure was judged appropriate by the athletes on the basis of their experienced cognitive and emotional responses. Perceived burden was assessed as a core component construct [23], through items addressing the perceived burden of capturing meal and snack occasions and management of the fiducial marker. Willingness for future use was treated as intent to continue to use, an established indicator of acceptability [21], and perceived behavioural reactivity was treated as a threat to the validity of the recorded intake rather than as a component of acceptability.

The questionnaire consisted of structured items assessing participant experiences with the mFR™ application and the associated image-capture protocol. Responses were recorded using a five-category ordinal Likert scale ranging from strongly disagree to strongly agree. The questionnaire evaluated perceived ease of image capture, ease of use of the application interface, technical performance, perceived burden associated with recording and with the fiducial marker, perceived behavioural reactivity, and willingness for future use.

Items were designed to capture participants’ perceptions of recording foods and beverages using image capture, including the ease of remembering to capture images before and after meals and snacks, the usability of the application interface and the perceived burden associated with including the fiducial marker. Additional items assessed whether participants perceived that dietary recording influenced their habitual eating behaviours or interfered with daily routines. Open-ended free-text response questions were included to allow participants to elaborate on perceived facilitators, barriers, and overall impressions of the dietary recording experience.

The combination of ordinal response items and open-ended free-text response questions allowed quantitative usability ratings to be complemented by participants’ own descriptions of their recording experience, consistent with methodological approaches previously applied in studies examining the usability of mobile food record systems [13,17].

### 2.6. Data Analysis

**Figure 3:**
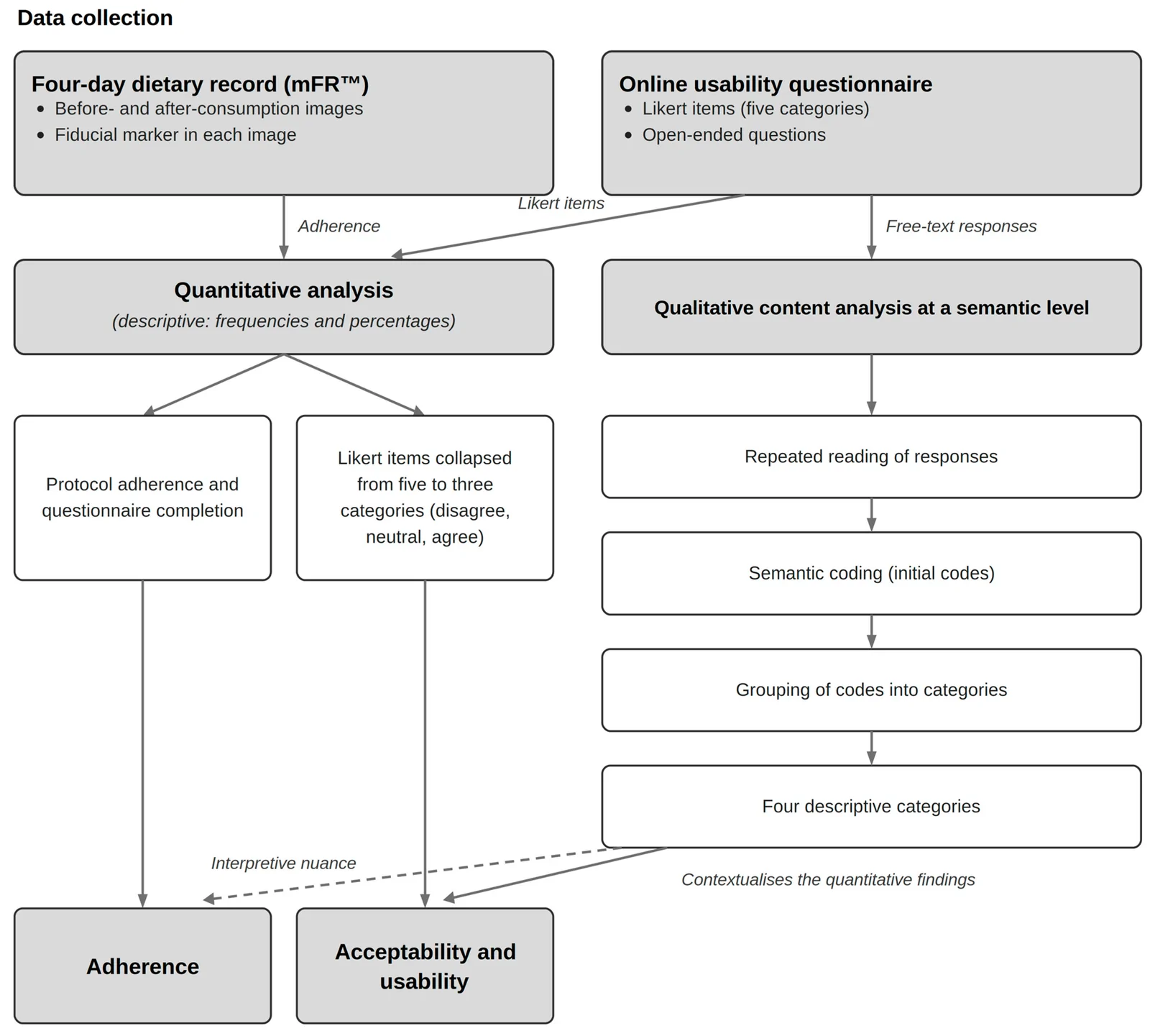
Overview of the data collection and analysis workflow. Quantitative data (protocol adherence and Likert-scale responses) were analysed descriptively and mapped to adherence, acceptability, and usability; open-ended responses were analysed using qualitative content analysis at a semantic level, producing four descriptive categories. mFR™ = mobile Food Record.

An overview of the data collection and analysis workflow is provided in Figure 3. Adherence to the dietary recording protocol was analysed descriptively. An eating occasion was defined as a distinct meal occasion recorded within a given day for which foods could be identified and analysed. A recording day was defined as any day on which at least one such eating occasion was recorded, including occasions entered through the “forgot image” function without images. A recording day therefore indicates that recording occurred on that day and does not establish that all eating occasions on that day were recorded. Recording days were ordered chronologically for each participant. Uploaded images were analysed by a single researcher (FF), who reviewed all before-eating and after-eating images and, drawing on the participant-added labels, identified each food and beverage and estimated portion sizes from the images, guided by the fiducial marker as a reference for size along with the visible geometry of plates, cups, and glasses, entering the corresponding food code and either an estimated weight or a standard household measure into nutrition analysis software (FoodWorks 10, Xyris Pty Ltd., Brisbane, Australia) linked to the AUSNUT 2011–13 food composition database [24]. EI is reported per recording day as recorded intake rather than as an estimate of habitual intake. Questionnaire responses to the Likert-scale items were initially recorded using five ordinal categories ranging from strongly disagree to strongly agree. However, for analytical purposes and to improve interpretability given the modest sample size and low frequencies observed in some response categories, these were collapsed into three categories: disagreement (strongly disagree and disagree), neutral, and agreement (agree and strongly agree). This decision was made during analysis and was not prespecified, and it follows the recoding applied to the same response scale in both source instruments [13,17]. The full five-category distributions are provided in Table S3. Given the exploratory nature of this study, quantitative usability and acceptability responses were analysed descriptively using frequencies and percentages in Microsoft Excel (Microsoft Corporation, Redmond, WA, USA). No inferential statistical comparisons were performed. No adherence threshold was prespecified, and adherence was interpreted in relation to prior evaluations of mobile food record systems in non-athlete populations [9,17].

The open-ended questions asked participants to describe what they liked most and least about the mFR™ app and to report any problems encountered while using it. Responses were analysed by one researcher (FF) using qualitative content analysis at a semantic level, informed by the coding principles described by Braun and Clarke [25], with categories developed inductively across the dataset rather than grouping responses under each open-ended question. The resulting descriptive categories were subsequently interpreted in relation to the study’s evaluation framework of acceptability and usability. Given the brevity of the free-text responses and the survey-based format, the analysis was descriptive and was intended to contextualise and elaborate the quantitative usability findings rather than to develop conceptually rich themes. Free-text responses were read repeatedly, segments carrying a single meaning were assigned an initial code, codes were compared across participants and grouped into descriptive categories, and category labels were refined until each accommodated all codes assigned to it. The resulting groupings are described as descriptive categories rather than as themes. Entries consisting only of “N/A” or equivalent were treated as non-responses and were not coded; no substantive response was excluded. Coding was undertaken by a single researcher, and no second coder and no assessment of coding reliability were applied, which is acknowledged as a limitation.

## 3. Results

### 3.1. Participant Characteristics

Twenty participants (mean ± standard deviation; age: 25.2 ± 7.6 y; body mass: 75.3 ± 13.0 kg; height: 174.8 ± 9.7 cm) were enrolled and completed the post-study questionnaire, comprising 13 males and 7 females (Table 2), and included individuals representing recreational, regional, and national levels of sport participation. Most participants (13 of 20, 65%) were classified as Tier 1 (recreationally active), with three classified as Tier 2 (trained/developmental) and four as Tier 3 (highly trained/national level) [18]. The mFR™ app was used on an iPhone by 17 participants (85%) and on an Android device by three (15%). Sports represented included resistance training (*n* = 5), triathlon (*n* = 4), basketball (*n* = 3), running (*n* = 2), and one participant each from jiu-jitsu, rowing, ultimate frisbee, field hockey, football (soccer), and cycling. With respect to training load, 15% of participants (*n* = 3) reported less than 6 h per week of sport-specific training, 55% (*n* = 11) reported 6 to 10 h, 25% (*n* = 5) reported 11 to 15 h, and 5% (*n* = 1) reported 16 to 20 h per week. Regarding non-sport-specific training, 60% (*n* = 12) reported 6 to 10 h per week, 35% (*n* = 7) less than 6 h per week, and one participant (5%) reported more than 11 h per week.

### 3.2. Adherence to the Recording Protocol

Adherence to the dietary recording request varied across the recording period. All 20 participants recorded on the first day, declining to 13 (65%) by the fourth day; of the seven participants who did not record on all four days, three recorded on three days, three on two days, and one on a single day. However, all participants completed the post-study usability questionnaire. The number of eating occasions captured and the recorded EI per recording day are presented in Table 3. Among those recording on a given day, a mean of approximately two eating occasions were captured (range 1 to 5), and recorded daily EI varied widely between participants. Descriptive indicators of the recorded eating occasions are presented in Table S2. The minimum values of 12 kJ on Days 1 and 2 were recorded by a single participant who captured one eating occasion on each of those days and are reported unaltered.

### 3.3. Perceptions on the Use of the Mobile Food Record

Table 1 shows the participant perceptions on the use of the mFR™ app. Most participants (85%, *n* = 17) agreed that they “found it easy to take photos of the food and beverages” using the mFR™ app. More than half of the participants (55%, *n* = 11) agreed that “remembering to take an image before meals was easy”, whereas only 15% (*n* = 3) agreed “remembering to take an image before snacks was easy”. Most participants (70%, *n* = 14) agreed that they found “the fiducial (checkerboard) marker easy to carry around”, and 80% (*n* = 16) agreed that “it is easy to label foods”. A total of 80% (*n* = 16) of participants agreed that “Understanding the purpose” of the app motivated them to use the app and 80% (*n* = 16) were “willing to undertake the TADA™ app again”.

The majority of participants (90%, *n* = 18) reported no problems when using the mFR™ app, as assessed by the corresponding questionnaire item. Two participants reported experiencing difficulties: one related to consistently carrying the fiducial marker when eating away from home, while the other experienced difficulty related to the overall image-capture and food-labelling workflow.

Half of participants (50%, *n* = 10) agreed that using the app “made me behave differently while I was using it”, whereas 35% (*n* = 7) disagreed and 15% (*n* = 3) were neutral.

### 3.4. Open-Ended Responses

Fourteen of the 20 participants (70%) provided at least one free-text response: 13 to the item asking what they liked most about the app, 8 to the item asking what they liked least, and 4 to the item inviting a description of any problems encountered, 2 of whom had also answered affirmatively to the preceding binary item. Blank responses and responses of “N/A” were treated as non-responses and no imputation was applied. Coding of these responses yielded four descriptive categories reflecting participants’ experiences of using the mFR™ app. The categories reflected perceived ease of use and usability; recording burden and practical challenges; food database limitations and desired enhancements; and behavioural responses and increased dietary awareness (Table 4).

Participants frequently described the application as intuitive, straightforward, and easy to use. Image capture and food labelling functions were generally perceived as user-friendly: “User friendly and no issues uploading the images” (recreational male athlete); “Easy to use—a quick process” (recreational male athlete). One participant described a reminder to capture the after-eating image as the most useful feature of the application: “The best feature was the reminder to take the after photo, I struggled to remember myself but the app would notify me” (recreational female athlete).

Despite overall positive perceptions of usability, participants reported several practical challenges. Difficulties remembering to capture snack occasions were commonly described, alongside perceptions that the recording process was occasionally time-consuming: “It was time consuming at times” (recreational female athlete); “sometimes, I was having a dinner with family or friends, and I need to stop to take the pictures and describe them. It is a time consuming!” (recreational male athlete). Participants also noted logistical barriers associated with managing the fiducial marker, particularly when eating away from home: “The square was a pain—I eat on the go a lot with my job and training, so couldn’t log my meals most times because I forgot to carry the square everywhere” (national female athlete); “I found it difficult to remember to bring the checkers board with me if I was eating out of home” (national female athlete).

Participants also described limitations related to the food database. Participants described difficulty locating specific foods or meals within the food list: “the list of foods I don’t think is detailed enough i.e., quarter of roast chicken was not listed, only listed as breast, thigh etc” (recreational female athlete); “Small range of food choices” (recreational male athlete). In addition, several participants suggested enhanced automation and more detailed nutritional feedback features: “I would prefer an app using AI to recognize food, and quantity with no need to take a photo after eating the food and describe what was that” (recreational male athlete); “I wish it gave me more information on what I was eating. Calories, fats etc” (national female athlete).

Some participants reported increased awareness of their eating behaviours during the recording period: “I have to think twice if what I was eating will be good enough for me. And take a time to consider to eat it or not” (recreational male athlete); “I liked that the app encouraged me to also track my food on a calorie counting app” (recreational female athlete). A minority described the recording process as occasionally intrusive during social eating situations.

## 4. Discussion

### 4.1. Overall Usability and Acceptability of the mFR™ App in Free-Living Athletes

Usability and acceptability of the mFR™ app were generally rated favourably by participants in this study. Thirteen of the 20 participants (65%) recorded at least one eating occasion on all four target days and all 20 completed the usability questionnaire. In the absence of a prespecified threshold for adequate adherence, these observations are reported descriptively and are not interpreted as establishing whether the recording protocol can be implemented in this population. Comparison of quantitative usability outcomes and qualitative findings indicated broadly consistent patterns across data sources, with generally positive perceptions of usability and high willingness to use the mFR™ app again (80%), although agreement that remembering to capture images was easy was more modest and occasion-dependent (55% and 60% for before- and after-meal capture, respectively, and markedly lower for snacks), corroborated by qualitative descriptions of the mFR™ app as intuitive and convenient. Qualitative responses also elaborated on specific challenges identified quantitatively, including difficulties with snack recording, fiducial marker management, and perceived recording burden, providing interpretative nuance that enriched the quantitative findings. The proportion of participants who recorded on all four days cannot be directly compared with prior evaluations of the mFR™ application in non-athlete populations [9,17]. Those evaluations were conducted under controlled conditions with one or two meals [17] or over 7.5 days among community-dwelling adults who were provided with meals and from whom individuals undertaking extreme forms of exercise were excluded, and among whom 40 of 45 provided images on every recording day [9]. The one image-based study in athletic individuals reported that 19 of 20 participants completed three days of recording, although recording was undertaken alongside a concurrent weighed food diary [14]. No benchmark exists for unsupervised image-based recording in athletes. All participants had used the mFR™ app in the preceding controlled feeding study, and this prior exposure may have favoured adherence relative to that of first-time users. The full questionnaire completion rate indicates that participants engaged with the post-recording evaluation.

Establishing whether accurate dietary assessment can be achieved in free-living athletes under real-world conditions is an essential prerequisite before advancing a novel dietary assessment tool into larger-scale research or applied monitoring programmes. Seven of the 20 participants did not record on all four days. Moreover, it could not be verified for any participant whether all eating occasions were captured on the days that were recorded. The reasons for non-adherence were not collected systematically. The free-text responses point to difficulty remembering to capture images, particularly for snacks and when eating away from home, and to the time required for recording, and it cannot be excluded that some participants stopped recording rather than alter what they ate, which would itself be a form of reactivity. Incomplete adherence over a period as short as four days is relevant for both research and applied practice, particularly where recorded intake is used to estimate energy availability, because the loss of recording days would be expected to increase over the longer monitoring periods required to characterise habitual intake. The application of image-based dietary assessment tools in field-based sports nutrition research may reduce reliance on memory-dependent reporting [7,10]. Usability and acceptability were rated favourably, while adherence was incomplete, and half of the participants reported that recording altered their eating behaviour. A method may therefore be acceptable to its users and, at the same time, alter the behaviour it is intended to measure, and acceptability should not be read as evidence of measurement adequacy. Any reduction in memory-related error does not, however, guarantee a reduction in overall misreporting, and the present findings indicate that omission of eating occasions and behavioural reactivity remain possible. Given the study’s exploratory nature and relatively small sample size, these observations should be interpreted cautiously and primarily as preliminary evidence for the potential application of the mFR™ app in athlete populations.

### 4.2. Perceived Usability and Acceptability of the Image-Based Dietary Assessment Method

Participants reported generally positive usability perceptions, indicating that capturing dietary intake using the mFR™ application was easy and practical across most eating occasions (Table 1). Participants agreed that capturing images of foods and beverages was easy, and the food-labelling process during the confirmation step was perceived as straightforward. These findings are consistent with previous evaluations of the mFR™ application and comparable image-based dietary assessment tools in non-athlete populations, in which ease of use has been identified as a key facilitator of sustained participant engagement [13,17]. Agreement with the ease of before-meal image capture (55%) and after-meal image capture, including leftovers (60%), further suggests that the structured image-capture workflow is perceived as manageable by most participants across most main-meal occasions.

Image-based dietary assessment tools may offer several methodological advantages over traditional estimated food diaries and 24 h dietary recalls. Real-time, in situ image capture reduces reliance on retrospective memory reconstruction, which has consistently been identified as a major source of measurement error in dietary assessment research [4]. The photographic record also provides a visual reference for analyst-assisted interpretation and portion-size estimation, reducing some of the uncertainty associated with written or verbal self-reports [15]. These attributes are particularly relevant in athletic populations, whose dietary patterns often involve frequent eating occasions, mixed meals, and sport-specific supplementation practices that are difficult to capture accurately using conventional methods [3,6]. The high proportion of participants expressing willingness to use the mFR™ app again (80%) indicates acceptability of the recording procedure and a stated intention to use it on a future occasion. Repeatability was not assessed, as this would require repeated administration of the method and evaluation of the consistency of estimates across occasions. Nevertheless, further research is required to determine whether these positive usability perceptions are maintained during longer monitoring periods or in populations with more complex sport-specific nutritional practices.

### 4.3. Practical Challenges Affecting Adherence and Data Completeness

Despite generally favourable usability perceptions, several practical challenges were identified that may meaningfully influence adherence and data completeness when the mFR™ app is implemented in free-living athletes. The primary challenge identified pertained to capturing images when snacking, with 60% of participants reporting difficulty capturing pre-snack images and 40% indicating comparable difficulties with post-snack image capture. This represents a pattern of perceived ease relative to main meal occasions. Qualitative responses elaborated on the broader challenge of remembering image capture. Participants described difficulties when eating away from home, on the move, or in informal and social eating situations. This pattern has important methodological implications for dietary assessment in athlete populations. Notably, Costello et al. [26] demonstrated that a photography-based dietary assessment method can yield valid and reliable EI estimates in elite adolescent athletes when implemented alongside a comprehensive behaviour change framework, suggesting that structured adherence-support strategies may be particularly important for image-based recording in this population. Snack occasions accounted for 34 of the 161 eating occasions recorded across the recording days (21%; Table S2). The number of snack occasions that went unrecorded cannot be quantified and omission cannot be demonstrated. Participants reported greater difficulty in remembering to capture snack images than meal images, and snacks constituted a minority of the occasions that were recorded. The same pattern was observed objectively when adolescents recorded with the mFR™ under supervision, with images captured for 90% of breakfasts and lunches but for only 54% of afternoon and 40% of evening snacks [11]. As athletes commonly consume multiple eating occasions throughout the day to support high energy expenditure, training recovery, and performance [1], any systematic non-recording of snack occasions would be expected to contribute to an underestimation of total daily energy and carbohydrate intake, particularly in athlete populations where snacks often represent an important component of daily EI.

Management of the fiducial marker also represented an additional logistical challenge. Although 70% of participants reported that the marker was easy to carry, qualitative responses indicated that consistently including the marker across all eating occasions was difficult, particularly when eating away from home or during periods of intensive training schedules. The requirement to carry and present a physical reference object introduces a degree of procedural burden that may be incompatible with the variable and often unpredictable eating environments characteristic of athletes.

A further barrier identified was limitations within the food database, with participants reporting difficulty locating sport-specific foods, mixed dishes, and composite meals within the available food list. Athletes commonly consume sport-specific foods and supplements that are frequently absent from generalised food composition databases, a limitation previously noted in dietary assessment research conducted in athletic populations [6]. Such limitations are not unique to the mFR™ in its current implementation; Stables et al. [27] reported that estimating complex mixed meals from food photographs introduced substantial error, even among experienced sport nutritionists, highlighting the compounding challenges of both missing foods in databases and analysts’ interpretation of photographed dishes. These findings suggest that although the core image-capture workflow is perceived as manageable, certain procedural and technical aspects of the current mFR™ implementation may contribute to incomplete dietary records under free-living conditions.

Future protocol refinements may therefore benefit from strategies designed to reduce snack-recording burden. The application used in this study did not deliver scheduled reminders to record, and the reminder mentioned by one participant in the free-text responses referred to the after-eating image. What is proposed here is prompting for eating occasions that have not been initiated, which the application did not provide. Prompts timed to each participant’s habitual eating times have been shown to increase the number of images captured in an image-based dietary record, whereas prompts at fixed times did not [28], which suggests that any such prompting would need to be tailored to the individual’s eating pattern. Expanding the food composition database to better reflect sport-specific dietary practices would address a separate barrier identified by participants. Standardised guidelines for optimising adherence to image-based dietary recording protocols in athletes have not yet been established [2,6], and this aspect of implementation warrants further investigation.

### 4.4. Behavioural Reactivity and Implications for Dietary Assessment Research

That 50% of participants reported dietary recording influenced their eating behaviour during the recording period highlights the potential for behavioural reactivity when monitoring dietary intake in athlete populations. Behavioural reactivity refers to the phenomenon whereby the act of monitoring induces measurable changes in the behaviour when under observation and is a recognised methodological limitation in self-report dietary assessment research [3]. Although participants were explicitly instructed to maintain their habitual dietary behaviours throughout the recording period, half reported that recording altered their dietary behaviour. This finding is broadly comparable with prior evaluations of the mFR™ app in community-dwelling adults, in which 69% of participants reported that dietary recording influenced their behaviour [9]. Free-text responses described increased awareness of food choices and, in one instance, reconsideration of what was eaten. These accounts are self-reported and were not accompanied by any objective measure of dietary change, so the direction and magnitude of any change in intake cannot be established from the present data. The pattern is nevertheless consistent with the behavioural reactivity previously described in dietary self-monitoring research [3].

The implications of behavioural reactivity for estimating habitual dietary intake in athletes warrant consideration. If dietary recording prompts individuals to alter food selection, meal frequency, or portion size, intake estimates may no longer accurately reflect habitual dietary behaviours or typical energy availability, which is a fundamental construct that underpins the evaluation of athlete health, training adaptation, and performance outcomes [1,8]. In addition, short monitoring periods may not fully capture the natural day-to-day variability in dietary intake that is characteristic of free-living athletes, an observation supported by prior work in elite road cyclists [8].

Whether longer monitoring periods permit behavioural habituation is a hypothesis that remains to be tested rather than an established solution, and it is complicated by the decline in adherence observed here over only four days. Future studies employing the mFR™ app or similar image-based dietary assessment tools should therefore examine reactivity and adherence jointly across longer monitoring periods and should incorporate strategies to assess reactivity systematically where possible. Evaluation should also be extended to ecologically demanding contexts such as competition or tournament settings, where recording burden and behavioural reactivity may differ substantially from routine training periods. The present findings provide preliminary support for the acceptability of the mFR™ system in athlete populations; however, the influence of behavioural reactivity on the accuracy of dietary intake estimates obtained through image-based methods warrants further investigation before definitive conclusions regarding their application in athlete monitoring can be drawn.

### 4.5. Limitations

Several limitations of this investigation should be acknowledged. First, the study included a relatively small sample of convenience, which may limit the transferability of findings to other athlete populations. Second, dietary recording was conducted over four consecutive days and may not fully capture habitual dietary intake or day-to-day variability in eating behaviours. In addition, adherence was assessed at the level of days on which at least one eating occasion was recorded rather than the completeness of recordings within each day, because participants’ habitual eating frequency was not captured; therefore, it could not be determined whether all eating occasions were recorded on a given day. Third, acceptability outcomes were based on self-reported perceptions, which should ideally be triangulated with observed behaviour and may have been influenced by social desirability or recall bias. Fourth, the study was designed to evaluate usability and acceptability rather than the validity of dietary intake estimates; accordingly, the recorded intake data are presented descriptively and should not be interpreted as accurate estimates of habitual intake. Fifth, no adherence threshold was prespecified, and no formal sample size justification was undertaken, so the observed adherence cannot be evaluated against a criterion defined in advance. Sixth, the sample was heterogeneous in sport and competitive level and was predominantly Tier 1, recreationally active, which limits generalisability to trained and elite athletes, to competition and travel contexts, and to longer monitoring periods. Seventh, participation required ownership of and familiarity with a smartphone, which may have selected individuals more comfortable with mobile technology and may therefore have yielded more favourable usability ratings than would be obtained in an unselected athlete population. Further to this familiarity point, all participants had used the mFR™ app under supervision in the preceding controlled feeding study before entering the present study. As such, the usability ratings and adherence reported here describe athletes with prior exposure to the application and may be more favourable than those of first-time users. Eighth, the adapted acceptability and usability questionnaire was not pilot tested in athletes and was not subjected to formal psychometric evaluation. Ninth, the qualitative component was coded by a single researcher without an assessment of coding reliability. Finally, favourable usability ratings coexisted with incomplete recording adherence, indicating that perceived usability and observed recording behaviour should be evaluated separately rather than treated as interchangeable.

### 4.6. Practical Recommendations

Sport scientists and nutrition practitioners seeking to implement image-based dietary assessment tools in free-living athletes should consider several protocol-level modifications informed by the present findings. First, targeted education and reminder strategies for snack occasions should be incorporated during participant onboarding, given that snack recording was identified as a challenge to adherence. Second, the burden associated with carrying the fiducial marker should be anticipated during onboarding, for example, by explaining its purpose and by supplying more than one marker so that one can be kept at each of the participant’s usual eating locations, a solution proposed by a participant in the present study. Third, given that behavioural reactivity was reported by approximately half of participants, practitioners should exercise caution when interpreting EI estimates derived from short monitoring periods (≤4 days). Energy availability cannot be represented by any single objective indicator, and the recommendation is therefore to interpret recorded intake alongside independent measures of the remaining components of the energy availability equation, such as objectively measured exercise energy expenditure and body composition assessed using a standardised protocol. Collectively, these considerations may improve data completeness, reduce participant burden, and enhance the interpretability of dietary intake estimates obtained using the mFR™ system in both applied and research settings.

## 5. Conclusions

Usability and acceptability of the mFR™ app were generally rated favourably by participants in this study, with most finding image capture straightforward, reporting no problems with the application and indicating willingness to use it again. Adherence to the four-day recording protocol was incomplete, declining from all 20 participants on the first day to 13 (65%) by the fourth, and the completeness of recording within each day could not be established. Snack occasions were the most frequently reported recording difficulty and accounted for a minority of the eating occasions recorded. Half of the participants reported that recording altered their eating behaviour. We therefore judge that the mFR™ app was generally acceptable to participants in this preliminary study, whereas its capacity to capture complete dietary records under free-living conditions remains to be demonstrated. Adherence support and verification of within-day completeness should be addressed before the method is applied to estimate habitual intake in this population.

## Figures and Tables

**Table 1:** Participant responses to the mFR™ usability and acceptability questionnaire.

Item	Disagree *n* (%)	Neutral *n* (%)	Agree *n* (%)
I found it easy to take photos of the food and beverages I ate or drank using the TADA™ app	3 (15)	0 (0)	17 (85)
Remembering to take an image BEFORE meals was easy	7 (35)	2 (10)	11 (55)
Remembering to take an image for leftovers AFTER meals was easy	5 (25)	3 (15)	12 (60)
Remembering to take an image BEFORE snacks was easy	12 (60)	5 (25)	3 (15)
Remembering to take an image for leftovers AFTER snacks was easy	8 (40)	7 (35)	5 (25)
I found the fiducial (checkerboard) marker easy to carry around	4 (20)	2 (10)	14 (70)
I found it easy to label my foods with coloured pins during the review process	3 (15)	1 (5)	16 (80)
I found it easy to find my foods in the food list	4 (20)	5 (25)	11 (55)
I found it easy to add foods using the “food not listed” function	3 (15)	6 (30)	11 (55)
I found it easy to record foods using the “forgot image” food list	2 (10)	7 (35)	11 (55)
Understanding the purpose of the TADA™ app motivated me to use it	1 (5)	3 (15)	16 (80)
Using the TADA™ app made me behave differently while I was using it	7 (35)	3 (15)	10 (50)
I would be willing to undertake the TADA™ app again	1 (5)	3 (15)	16 (80)

Note. Values are percentages of respondents (*n* = 20). Responses were collapsed from five ordinal categories into three: disagreement (strongly disagree and disagree), neutral, and agreement (agree and strongly agree). All items answered on the five-point agreement scale are presented; items with binary or frequency response formats and the open-ended questions are provided in Table S1. Item wording is reproduced as administered; “the TADA app” in the item text refers to the mFR™ participant-facing interface of the TADA™ system.

**Table 2:** Sex, age, anthropometry, and weekly training volume, overall and by participant classification tier.

Characteristic	Total (*n* = 20)	Tier 1 (Recreationally Active)	Tier 2 (Trained/Developmental)	Tier 3 (Highly Trained/National Level)
Male	13	9	2	2
Female	7	4	1	2
Age (y)	25.2 ± 7.6	26.1 ± 8.6	26.3 ± 8.0	21.7 ± 2.5
Body mass (kg)	75.3 ± 13.0	74.8 ± 13.7	83.1 ± 16.7	71.4 ± 7.7
Height (cm)	174.8 ± 9.7	173.4 ± 9.4	184.1 ± 12.4	172.5 ± 6.3
BMI (kg·m^−2^)	24.5 ± 3.0	24.7 ± 3.2	24.3 ± 2.5	24.1 ± 3.4
**Sport-specific training (h·wk^−1^)**				
<6 h·wk^−1^	3	2	1	0
6–10 h·wk^−1^	11	9	0	2
11–15 h·wk^−1^	5	2	1	2
16–20 h·wk^−1^	1	0	1	0
**Non-sport-specific training (h·wk^−1^)**				
<6 h·wk^−1^	7	—	—	—
6–10 h·wk^−1^	12	—	—	—
>11 h·wk^−1^	1	—	—	—

Note. Values are presented as mean ± SD for continuous variables and as counts for categorical variables. Participant classification tiers are based on the Participant Classification Framework [18]. “Non-sport-specific training” refers to general strength and conditioning training performed in addition to the primary sport. BMI = body mass index.

**Table 3:** Descriptive summary of recorded dietary intake data, per recording day.

	Day 1	Day 2	Day 3	Day 4
**Participants (*n*)**	20	19	16	13
**Eating occasions (*n*/day)**				
Mean	2.5	2.3	2.4	2.2
SD	1.1	1.2	0.8	1.1
Max	4	5	4	4
Min	1	1	1	1
**Recorded energy intake (kJ/day)**				
Mean	4994	4648	4566	4455
SD	2718	3581	1824	2856
Max	9568	15,840	7452	11,603
Min	12	12	962	863

Note. Days are ordered chronologically for each participant. A recording day is a day on which at least one eating occasion was recorded and does not indicate that all eating occasions on that day were recorded. Values describe the intake that was recorded and analysed.

**Table 4:** Development of descriptive categories from initial codes generated from open-ended questionnaire responses.

Descriptive Category	Initial Code	Illustrative Participant Quote
Perceived ease of use and usability	Ease of use	“Easy, quick to use” (P23)
	Positive usability perception	“Easy to long [sic] and enjoyed pinning and tagging components of the meal” (P15)
	Convenience	“Convenience” (P32)
	Reminder usefulness	“The best feature was the reminder to take the after photo, I struggled to remember myself but the app would notify me” (P16)
Recording burden and practical challenges	Time burden	“It was time consuming at times” (P8)
	Recording burden	“After taking the picture, writing what exactly was [eaten] took time as well” (P50)
	Fiducial marker burden	“I found it difficult to remember to bring the checkers board with me if I was eating out of home” (P38)
	Marker misplacement concern	“Having more than one colour panel would be good for multiple sites… would help with misplacing it” (P6)
	Social interference	“Sometimes, I was having a dinner with family or friends, and I need to stop to take the pictures…” (P50)
Food database limitations and desired enhancements	Food database limitation	“The list of foods I don’t think is detailed enough i.e., quarter of roast chicken was not listed…” (P16)
	Desire for enhanced nutritional feedback	“I wish it gave me more information on what I was eating. Calories, fats etc” (P38)
	Desire for automation or AI	“I would prefer an app using AI to recognize food, and quantity with no need to take a photo after eating…” (P50)
	Feature discoverability issue	“I was unaware there was an option for food not listed” (P16)
	Feature awareness issue	“Didn’t realise there was an option to select foods not listed when labelling” (P23)
Behavioural responses and increased dietary awareness	Positive behavioural influence	“I liked that the app encouraged me to also track my food on a calorie counting app” (P8)
	Behavioural awareness	“I have to think twice if what I was eating will be good enough for me. And take a time to consider to eat it or not” (P50)

Note. Open-ended responses were analysed using qualitative content analysis at a semantic level [25]. Initial codes were generated inductively from the free-text responses of 14 participants and grouped into four descriptive categories. Quotes are presented verbatim and are illustrative rather than exhaustive; square brackets indicate text added for clarity, ellipses indicate omitted text and [sic] denotes an original error. Participant identifiers (P) correspond to the study identification numbers.

## Data Availability

The data presented in this study are available on request from the corresponding author due to privacy restrictions.

## Supplementary Materials

The following supporting information can be downloaded at: https://www.mdpi.com/article/10.3390/nu18193208/s1, Table S1: Acceptability and usability questionnaire; Table S2: Descriptive summary of image capture and recorded eating occasions; Table S3: Full five-category response distributions for the acceptability and usability questionnaire.

## Abbreviations

The following abbreviations are used in this manuscript:

EA	energy availability
EI	energy intake
mFR™	mobile Food Record
PIF	Participant Information Form
SD	standard deviation
TADA™	Technology-Assisted Dietary Assessment
