# Supplementary material for: Usability and Acceptability of a Mobile Food Record in Free-Living Athletes: A Single-Group Observational Study
Source: Nutrients. 2026 Sep 28;18(19):3208. doi: 10.3390/nu18193208 (PMC13666683; doi:10.3390/nu18193208)
Supplement: Supplementary file 1 [file nutrients-18-03208-s001.zip › nutrients-4545964-supplementary.pdf]

Supplementary Material

Table S1. Acceptability and usability questionnaire.

No.	Item	Response options
1	What mobile were you using for the TADA app?	iPhone; Android
2	I found it easy to take photos of the food and beverages I ate or drank using the TADA App.	5-point agreement scale [a]
3	Remembering to take an image BEFORE MEALS was easy.	5-point agreement scale [a]
4	Remembering to take an image for leftovers AFTER MEALS was easy.	5-point agreement scale [a]
5	Remembering to take an image BEFORE SNACKS was easy.	5-point agreement scale [a]
6	Remembering to take an image for leftovers AFTER SNACKS was easy.	5-point agreement scale [a]
7	I found the fiducial (checkerboard) marker easy to carry around.	5-point agreement scale [a]
8	I found it easy to label my foods with the coloured pins during the review process.	5-point agreement scale [a]
9	I found it easy to find my foods in the food list.	5-point agreement scale [a]
10	I found it easy to add my foods using the “food not listed” function.	5-point agreement scale [a]
11	I found it easy to record my foods in the “forgot image” food list.	5-point agreement scale [a]
12	I had problems using the TADA app.	Yes, I had problems; No, I didn’t have problems
13	If you had any problems with the TADA app, please describe the problems you had.	Open-ended response
14	Understanding the purpose of the TADA app motivated me to use it.	5-point agreement scale [a]
15	Using the TADA app made me behave differently while I was using it.	5-point agreement scale [a]
16	I would be willing to undertake the TADA app again.	5-point agreement scale [a]
17	List what you liked most (if anything) about the TADA app.	Open-ended response
18	List what you liked least (if anything) about the TADA app.	Open-ended response
19	Do you take photos of your food?	No, I never take photos of my food; Yes, I take photos of my food
20	I take photos of my food.	Frequency scale (Never; Rarely; Sometimes; Often; Very often) rated for each context: Eating out; At home; Other (please state) [b]

[a] 5-point agreement scale: Strongly disagree; Disagree; Neither agree nor disagree; Agree; Strongly agree.

[b] Item 20 was administered as a frequency matrix.

Items are numbered consecutively from 1 to 20. The questionnaire as administered in Qualtrics used a non-consecutive numbering sequence; no items were added or removed in renumbering. Administrative fields collected before item 1 (first name, last name) are not shown.

Table S2. Descriptive summary of image capture and recorded eating occasions.

(a) Image capture, per participant.

PID	Recording days (n)	Images (n)	Before-and-after image pairs (n)	Pairs in which the after-eating image showed the fiducial marker only (n)	Pairs in which the before-eating image showed the fiducial marker only (n)	Images in which the fiducial marker was present (n)	"Forgot image" entries (n)	Eating occasions analysed (n)
1	4	22	11	2	0	22	0	11
4	2	4	2	0	0	4	0	2
6	3	32	16	0	0	32	0	9
8	3	20	10	0	0	20	0	7
10	4	18	9	0	0	18	0	8
11	4	10	5	0	0	10	0	5
13	4	10	5	0	1	10	0	8
14	2	8	4	0	0	6	0	3
15	4	22	11	0	0	22	0	10
16	4	18	9	0	0	18	0	9
23	4	10	5	0	0	10	5	8
28	3	10	5	0	0	10	0	6
32	4	12	6	0	0	12	1	7
35	2	4	2	0	0	4	0	2
37	4	22	11	1	0	22	0	11
38	4	24	12	6	6	12	0	9
43	1	2	1	0	0	0	3	3
50	4	54	27	1	1	52	0	16
51	4	10	5	0	0	10	10	11
52	4	24	12	0	0	24	22	16
**Total**	**68**	**336**	**168**	**10**	**8**	**318**	**41**	**161**

(b) Recorded eating occasions, per recording day.

	Day 1	Day 2	Day 3	Day 4	Total
Participants recording (n)	20	19	16	13	
Eating occasions analysed (n)	50	44	38	29	161
Food items per eating occasion (mean ± SD)	3.1 ± 1.8	2.5 ± 1.4	2.6 ± 1.3	2.9 ± 1.4	2.8 ± 1.5
Eating occasions by type (n)					
Breakfast	13	12	11	9	45 (28%)
Lunch	11	8	11	7	37 (23%)
Dinner	17	12	9	7	45 (28%)
Snack	9	12	7	6	34 (21%)

Image counts in panel (a) were obtained from the application records for each participant and cover all recording days. A recording day is a day on which at least one eating occasion was recorded and does not indicate that all eating occasions on that day were recorded. Eating occasions analysed comprise occasions documented by images and occasions reported through the "forgot image" function, in which the participant entered the food, the time of consumption and an approximate amount without images; both were identified and quantified in the dietary analysis. An image showing the fiducial marker only is one in which the participant photographed the marker without food or beverages, for example when nothing remained after eating; such images are included in the count of images in which the marker was present. PID = participant identifier; SD = standard deviation.

Table S3. Full five-category response distributions for the acceptability and usability questionnaire.

No.	Item	Strongly disagree	Disagree	Neither agree nor disagree	Agree	Strongly agree
2	I found it easy to take photos of the food and beverages I ate or drank using the TADA App.	1	2	0	11	6
3	Remembering to take an image BEFORE MEALS was easy.	2	5	2	10	1
4	Remembering to take an image for leftovers AFTER MEALS was easy.	0	5	3	9	3
5	Remembering to take an image BEFORE SNACKS was easy.	5	7	5	1	2
6	Remembering to take an image for leftovers AFTER SNACKS was easy.	2	6	7	4	1
7	I found the fiducial (checkerboard) marker easy to carry around.	0	4	2	10	4
8	I found it easy to label my foods with the coloured pins during the review process.	0	3	1	10	6
9	I found it easy to find my foods in the food list.	0	4	5	7	4
10	I found it easy to add my foods using the “food not listed” function.	0	3	6	8	3
11	I found it easy to record my foods in the “forgot image” food list.	0	2	7	8	3
14	Understanding the purpose of the TADA app motivated me to use it.	0	1	3	14	2
15	Using the TADA app made me behave differently while I was using it.	0	7	3	7	3
16	I would be willing to undertake the TADA app again.	1	0	3	12	4

Values are counts of respondents. Item numbers and wording correspond to Table S1; item wording is reproduced as administered, in which the application was referred to as “the TADA app”. Table 1 in the main text reports the same items with the five categories collapsed to three (disagreement, neutral, agreement).
